# The Temporal and Hierarchical Control of Transcription Factors-Induced Liver to Pancreas Transdifferentiation

**DOI:** 10.1371/journal.pone.0087812

**Published:** 2014-02-04

**Authors:** Dana Berneman-Zeitouni, Kfir Molakandov, Marina Elgart, Eytan Mor, Alessia Fornoni, Miriam Ramírez Domínguez, Julie Kerr-Conte, Michael Ott, Irit Meivar-Levy, Sarah Ferber

**Affiliations:** 1 Sheba Regenerative Medicine, Stem cells and Tissue engineering Center, Sheba Medical Center, Tel-Hashomer, Israel; 2 Department of Human Genetics and Molecular Medicine, Sackler School of Medicine, Tel-Aviv University, Tel-Aviv, Israel; 3 Rabin Medical Ctr., Beilinson Campus, Petah-Tiqva, Israel; 4 Diabetes Research Institute, University of Miami Leonard M. Miller School of Medicine, Miami, Florida, United States of America; 5 University Lille Nord de France, Lille, France; 6 Gastroenterology, Hepatology and Endocrinology, Hannover Medical School, Germany; Twincore, Centre for Experimental and Clinical Infection Research, Hannover, Germany; National University of Singapore, Singapore

## Abstract

Lineage-specific transcription factors (TFs) display instructive roles in directly reprogramming adult cells into alternate developmental fates, in a process known as transdifferentiation. The present study analyses the hypothesis that despite being fast, transdifferentiation does not occur in one step but is rather a consecutive and hierarchical process. Using ectopic expression of Pdx1 in human liver cells, we demonstrate that while glugacon and somatostatin expression initiates within a day, insulin gene expression becomes evident only 2–3 days later. To both increase transdifferentiation efficiency and analyze whether the process indeed display consecutive and hierarchical characteristics, adult human liver cells were treated by three pancreatic transcription factors, Pdx1, Pax4 and Mafa (3pTFs) that control distinct hierarchical stages of pancreatic development in the embryo. Ectopic expression of the 3pTFs in human liver cells, increased the transdifferentiation yield, manifested by 300% increase in the number of insulin positive cells, compared to each of the ectopic factors alone. However, only when the 3pTFs were sequentially supplemented one day apart from each other in a direct hierarchical manner, the transdifferentiated cells displayed increased mature β-cell-like characteristics. Ectopic expression of Pdx1 followed by Pax4 on the 2^nd^ day and concluded by Mafa on the 3^rd^ day resulted in increased yield of transdifferentiation that was associated by increased glucose regulated c-peptide secretion. By contrast, concerted or sequential administration of the ectopic 3pTFs in an indirect hierarchical mode resulted in the generation of insulin and somatostatin co-producing cells and diminished glucose regulated processed insulin secretion. In conclusion transcription factors induced liver to pancreas transdifferentiation is a progressive and hierarchical process. It is reasonable to assume that this characteristic is general to wide ranges of tissues. Therefore, our findings could facilitate the development of cell replacement therapy modalities for many degenerative diseases including diabetes.

## Introduction

Cell replacement therapies have been suggested as promising approaches for treating numerous degenerative diseases [1], [2]. Direct adult cell reprogramming or transdifferentiation could represent an alternative strategy for cellular therapies. Transdifferentiation is the direct conversion of one type of adult cell into an alternate type of cell with a different function [3]. Lineage-specific transcription factors (TFs) have been suggested to display instructive roles in converting adult cells to endocrine pancreatic cells [4]–[7], neurons [8]–[10], hematopoietic cells [11] and cardiomyocyte lineages [12], suggesting that transdifferentiation can be induced between a wide spectrum of tissues.

Transdifferentiation into endocrine pancreatic cells is a long lasting process which persists long after the expression of the ectopically introduced TFs diminishes [4], [13], [14]. This is due to the activation of numerous specific, otherwise silent, TFs which initially collaborate with the ectopic factors to promote the alternate desired repertoire [15]–[18]. The host repertoire of genes is being turned off, while the alternate desired repertoire is being activated, without dedifferentiating into a “stemness”-like state [19].

As opposed to embryonic organogenesis, transdifferentiation is fast and occurs within a few days [16], [19]–[22]. However, little is known about the actual steps undertaken by a cell as it changes identity. A major, yet unaddressed question is whether transdifferentiation occurs in one step or rather is a progressive process, and to what extent it resembles hierarchical embryonic organogenesis. Thus, the temporal and hierarchical control of transdifferentiation is being presently analyzed using the *in vitro* experimental system of human liver to pancreas transdifferentiation.

Pancreas organogenesis is initiated by the homeobox transcription factor Pdx1, which is also required for β cell function in adults [23], [24]. The endocrine differentiation is then mediated by the basic helix–loop–helix factors Ngn3 [25] and NeuroD1 [26]. The paired homeobox factors Pax6 and Pax4 and Arx, have been implicated as key factors in the segregation of the different endocrine cell types [27], [28]. The final maturation along the β cell lineage and function is attributed to selective expression of Mafa in β cells in the adult pancreas [29]. Artificial alterations in the hierarchical expression of pancreatic transcription factors mediating pancreas organogenesis resulted in ablated pancreatic development and subsequent malfunction [30]–[33].

Using an *in vitro* experimental system of adult human liver cells, we previously demonstrated that Pdx1 activates the expression of numerous β cell and other pancreatic endocrine specific markers. It activates the expression of numerous key endogenous pTFs and induces glucose-regulated secretion of processed insulin [16], [19]–[22]. The present study analyzes the hypothesis that if indeed transdifferentiation is progressive and hierarchical, the sequence of the ectopically introduced TFs may affect the final outcome of the process. This in turn could be manifested by the segregation of the different pancreatic endocrine lineages and by the level of transdifferentiated cells maturation along the desired β cell lineage.

Indeed, our data demonstrate for the first time that liver to pancreas transdifferentiation is a sequential and progressive process. While combined ectopic pTFs expression increases transdifferentiation efficiency compared to individual pTFs, the maturation of the transdifferentiated cells along the desired β-like-lineage occurs only when the ectopic pTFs are sequentially delivered in a direct hierarchical mode mimicking the developmental sequence. Our study suggests a mechanistic explanation for the temporal control of the transdifferentiation process of liver to the endocrine pancreas cells. These mechanistic characteristics could be extended to transdifferentiation between additional tissues, to promote the curative potential of cell replacement therapy modalities for many degenerative diseases including diabetes.

## Materials and Methods

### Human Liver Cells

Adult human liver tissues were obtained from 10 different liver specimens taken from individuals 3–23 years old. Liver tissues were used with the approval from the Sheba Medical Center Committee on Clinical Investigations (the institutional review board, informed consent was written). All donors or guardians on the behalf of the minors/children participants provided written informed consent for the collection of all samples and subsequent analysis. The isolation of human liver cells was performed as described [16], [19]. The cells were cultured in Dulbecco’s minimal essential medium (1 g/l of glucose) supplemented with 10% fetal calf serum, 100 units/ml penicillin; 100 ng/ml streptomycin; 250 ng/ml amphotericin B (*Biological Industries, Beit Haemek*, Israel), and cultured at 37°C in a humidified atmosphere of 5% CO_2_ and 95% air.

### Viral Infection

The adenoviruses used in this study were as follows: *Ad-CMV-Pdx1* [16], [19], *Ad-RIP-luciferase* [34], *Ad-CMV-β-Gal*, *Ad-CMV-Mafa* (generous gift from Newgard, C.B., Duke University), *Ad-CMV-Pax4-IRES-GFP* (generous gift from St Onge, L. DeveloGen AG, Göttingen, Germany), and *Ad-CMV-Isl1* (generous gift from Kieffer, T. University of British Columbia, Vancouver, Canada). The viral particles were generated by the standard protocol [35].

Liver cells were infected with recombinant adenoviruses for 5–6 days supplemented with EGF (20 ng/ml; Cytolab, Ltd., Israel) and nicotinamide (10 mM; Sigma). The optimal multiplicity of infection (MOI) was determined according to cell survival (<75%) and induction of c-peptide secretion. The MOI of the viruses used were: *Ad-CMV-Pdx1* (1000MOI), *Ad-CMV-Pax4-IRES-GFP* (100MOI*)*, *Ad-CMV-Mafa* (10MOI) and *Ad-CMV* ***-*** *Isl1* (1 and 100 MOI).

### RNA Isolation, RT and RT-PCR Reactions

Total RNA was isolated and cDNA was prepared and amplified, as described previously [13], [16]. Quantitative real-time RT-PCR was performed using ABI Step one plus sequence Detection system (*Applied Biosystems*, CA, USA), as described previously [16], [19], [20]. The primer sets used in this study are listed in Table S1.

### C-peptide and Insulin Secretion Detection

C-peptide and insulin secretion were measured by static incubations of primary cultures of adult liver cells 6 days after the initial exposure to the viral treatment, as described [16], [19], [20]. The glucose-regulated c-peptide secretion was measured at 17.5 Mm glucose, which was determined by dose-dependent analyses to maximally induce insulin secretion from transdifferentiated liver cells, without having adverse effects on treated cells’ function [16], [19], [20]. C-peptide secretion was detected by radioimmunoassay using the human C-peptide radioimmunoassay kit (Linco Research, St. Charles, MO; <4% cross-reactivity to human proinsulin). Insulin (and or pro-insulin) secretion was detected by radioimmunoassay using the human insulin radioimmunoassay kit (DPC, Los- Angeles, CA; 32% cross-reactivity to human proinsulin). The secretion was normalized to the total cellular protein measured by a Bio-Rad protein assay kit.

### Luciferase Assay

Human liver cells were co-infected with *Ad-RIP-luciferase* (200MOI) and the various adenoviruses (as described below). Six days later, luciferase activity was measured using the Luciferase assay System (Promega, Madison, WI) and the LKB 1250 Luminometer (LKB, Finland). The results were normalized to total cellular protein measured by the Bio-Rad Protein Assay kit (Bio-Rad, Hercules, CA).

### Immunofluorescence

Human liver cells treated with the various adenoviruses, were plated on glass cover slides in 12-well culture plates (2×10^4^ cells/well). 4–5 days later, the cells were fixed and stained as described [16], [19], [20]. The antibodies used in this study were: anti-rabbit Pdx1, anti- goat Pdx1 (both 1∶1000 a generous gift from C.V. E. Wright), anti-human insulin, anti –human somatostatin (both 1∶100, Dako, Glostrup, Denmark), anti-Pax4 (1∶100; R&D Systems, Minneapolis, MN), anti-Mafa (1∶160; Santa Cruz Biotechnology, Inc., Santa Cruz, CA). The secondary antibodies used were: anti-rabbit IgG Cyanine (cy2) conjugated antibody 1∶250, anti-rabbit IgG indocarbocyanine (cy3) conjugated antibody 1∶250, anti-goat IgG Cyanine (cy2) conjugated antibody 1∶200, anti-goat IgG indocarbocyanine (cy3) conjugated antibody 1∶250, and anti-mouse IgG indocarbocyanine (cy3) conjugated antibody 1∶250 (all from *Jackson ImmunoResearch*, PA). Finally, the cells were stained with 4′, 6-diamidino-2-phenyl-indole (DAPI, *Sigma*). The slides were imaged and analyzed using a fluorescent microscope (Provis, Olympus).

### Statistical Analyses

Statistical analyses were performed with a 2-sample Student’s *t-*test assuming unequal variances.

## Results

### Pdx1-induced Liver to Pancreas Transdifferentiation is a Sequential and Consecutive Process

**Figure 1:**
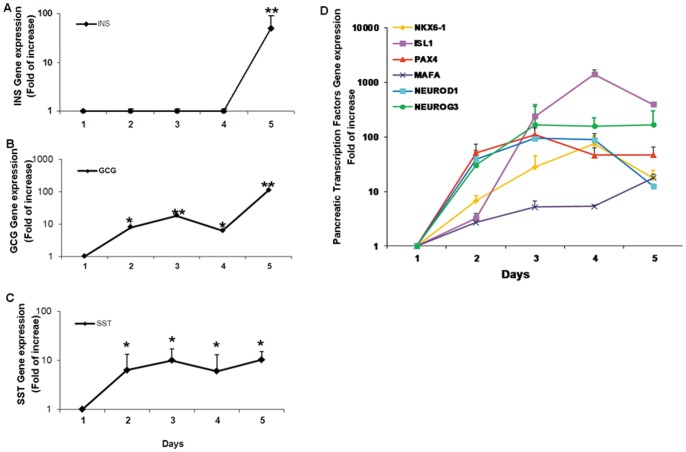
Pdx1 activates a sequential developmental process in adult human liver cells *in vitro*. Cultured adult human liver cells were infected with *Ad-CMV-Pdx1* (1000 MOI) or control virus (*Ad-CMV-β-gal*, 1000 MOI), and analyzed by quantitative RT-PCR every day for five days. **(A–C)** Expression of the pancreatic hormones and **(D)** the pTFs genes. The results were normalized to β-actin gene expression within the same cDNA sample and are presented as the mean ± SE of the relative expression versus control virus treated cells on the same day. n ≥4 in 2 independent experiments preformed in cells isolated from different donors. *p<0.05, **p<0.01.

Previous studies suggest that liver to endocrine pancreas transdifferentiation is fast and occurs within a few days [16], [19]–[22]. To analyze the sequence of events that mediate the process, we followed the activation of the pancreatic hormones gene expression during four consecutive days post adenoviral-mediated ectopic Pdx1 expression, in adult human liver cells *in vitro* (Days 2–5; Figure 1). The activation of the expression of different pancreatic hormones displayed distinct time course; while both glucagon and somatostatin genes were immediately activated, within one day after Ad-Pdx1 infection (Figure 1 B&C), insulin expression initiated two to three days later (Figure 1A). The activation of the pancreatic hormones in the liver results from a collaboration between the ectopic Pdx1 with numerous otherwise silent endogenous pTFs activated by the ectopic factor [16].

Therefore, we followed the activation of the endogenous pTFs expression during the Pdx1-induced transdifferentiation process. The activation of the expression of endogenous pTFs was also temporally distinct (Figure 1D). The early pancreatic endocrine transcription factors, NEUROG3 and NEUROD1 were immediately activated on the first day. ISL1 expression robustly increased between the second and third day but decreased on the fifth day (Figure 1D). By contrast, the late and β-cell specific TFs, NKX6.1 and MAFA, were only gradually activated in response to ectopic Pdx1 expression, reaching a peak on the fourth and fifth day, respectively. This suggests that transdifferentiation of human liver cells along the pancreatic lineage, despite being fast, is a progressive and consecutive process. Moreover, the distinct temporal activation of the expression of pancreatic hormones genes could be partially attributed to the time course and the relative levels of the endogenously activated pTFs in human liver cells.

### Combined Expression of Pdx1, Pax4 and Mafa Increases the Efficiency of Liver to Pancreas Transdifferentiation

**Figure 2:**
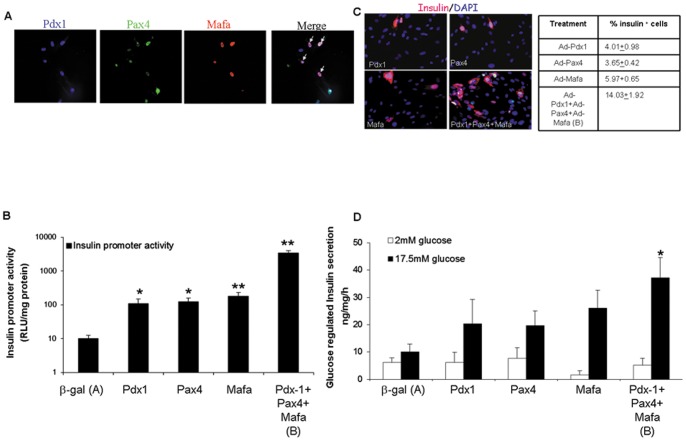
Ectopic co-expression of pTFs in the liver *in vitro* promotes endocrine pancreatic differentiation. Cultured adult human liver cells were infected with *Ad-CMV-Pdx1* (1000 MOI), *Ad-CMV-Pax4* (100 MOI) and *Ad-CMV-Mafa* (10 MOI) alone or in concert or with control virus (*Ad-CMV-β-gal*, 1000 MOI), and pancreatic differentiation markers were examined six days later. **(A)** Immunofluorescence staining of human liver cells ectopically treated with a combination of three pTFs; Pdx1 (blue), Pax4 (green), and Mafa (red). Arrows indicate cells positive for all three pTFs. **(B)** Cultures were co-infected with the combined pTFs and with *Ad-RIP-LUC* (200 MOI), and Luciferase activity was measured. The results are expressed as Relative Light Unit (RLU)/mg protein. Each data point represents the mean ± SE of at least 2 independent experiments preformed in cells isolated from different donors, *p<0.05, **p<0.01 in comparison to control virus treated cells (n>4). **(C)** Immunofluorescence staining of treated human liver cells for insulin (red). Nuclei were stained with DAPI (blue), original magnification X20. The percent of insulin-positive cells was calculated by counting at least 500 positive cells from at least 2 independent experiments preformed in cells isolated from different donors. **(D)** Insulin (and or pro-insulin) secretion was measured by static incubation of the cells for 15 min at 2 and 17.5 mM glucose in KRB. n>12 in 5 independent experiments preformed in cells isolated from different donors, *p<0.05 comparing between triple infection and all other treatments.

Numerous studies suggested that the concerted expression of several pTFs increases the transdifferentiation efficiency along the β-cell lineage, compared to that induced by an individual pTF [18], [21], [36]–[38]. In order to analyze this notion in primary adult human liver cell cultures, we compared the individual and joint effect of three major pTFs on liver to pancreas transdifferentiation. Pdx1, Pax4 and Mafa which mediate different stages in pancreas organogenesis, were ectopically expressed using recombinant adenoviruses. The multiplicity of infection (MOI) of each factor was titrated to result in maximal reprogramming efficiency which is associated by minimal adverse effects on infected cell viability. Each of the factors was expressed in about 50–60% of the cells in culture, and their joint co-expression was evident in 29% of the cells (Figure 2A). The combined expression of the three pTFs resulted in a substantial increase in insulin promoter activation over several orders of magnitude (Figure 2B, logarithmic scale). The number of insulin producing cells (Figure 2C) increased by 300%. All the insulin expressing cells co-expressed also the ectopic pTFs (Figure S1). Moreover, glucose-regulated insulin secretion (Figure 2D) was 30–60% higher than the secretion induced by each of the individual pTFs.

These results suggest that ectopic expression of individual pTFs is insufficient to induce a maximally efficient transdifferentiation process. This insufficiency is most probably caused by insufficient or improper activation of endogenous pTFs needed to complement each other in promoting an efficient transdifferentiation process [36], [37], [39].

### Maturation of Transdifferentiated Cells is a Temporally Controlled Process

To further analyze whether transdifferentiation is a progressive process as suggested above (Figure 1), we analyzed the impact of temporally and sequentially controlling the administration of ectopic pTFs.

**Figure 3:**
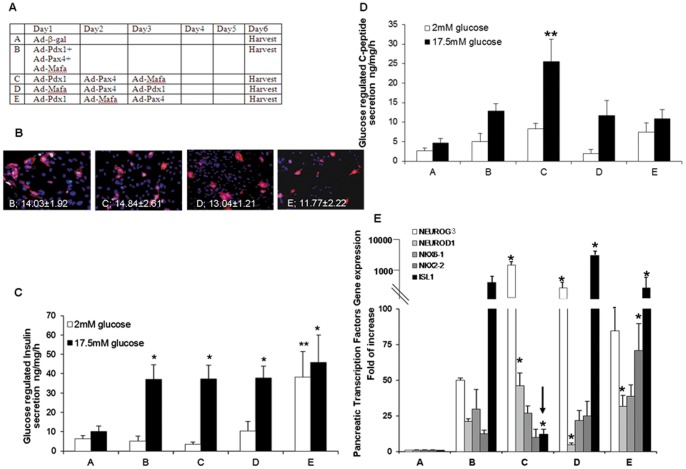
Combined expression of pTFs promotes transdifferentiation efficiency, however only their sequential expression increases β-cell maturation. Cultured adult human liver cells were infected with *Ad-CMV-Pdx1* (1000 MOI), *Ad-CMV-Pax-4* (100 MOI) and *Ad-CMV-Mafa* (10 MOI) together or in a sequential manner as summarized in (A) (treatments B–E) or with control virus (*Ad-CMV-β-gal*, 1000 MOI, treatment A), and analyzed for their pancreatic differentiation six days later. (B) Immunofluorescence staining of treated human liver cells for insulin (red). Nuclei were stained with DAPI (blue), original magnification X20. The percent of insulin positive cells were calculated by counting at least 1000 positive cells from at least two independent experiments preformed in cells isolated from different donors. Insulin (and or pro-insulin) (C) and c-peptide (D) secretion were measured by static incubation of the cells for 15 min at 2 and 17.5 mM glucose in KRB. *p<0.05, **p<0.01, compared to control virus treated cells; n≥12 in 5 independent experiments preformed in cells isolated from different donors. (E) Quantitative Real-Time PCR analysis for gene expression of endogenous pTFs. CT values are normalized to β-actin gene expression within the same cDNA sample. Results are presented as relative levels of the mean±SE of the relative expression versus control virus treated cells. *p<0.05, n≥8 in 4 independent experiments preformed in cells isolated from different donors. The arrow points to the specific decrease in Isl1 expression level under the C-protocol, sequential and direct hierarchical administration of pTFs.

The three pTFs (Pdx1, Pax4, and Mafa) which mediate distinct stages of pancreas organogenesis were introduced in concert or sequentially, one day apart from each other, to primary cultures of adult human liver cells using recombinant adenoviruses. Adenovirus-mediated ectopic gene expression peaks 17 hours post infection [40]. Therefore, the separate sequential administration of each of the pTFs during three consecutive days (Figure 3A), allows the manifestation of their individual effects. We infected the cells in a direct hierarchical mode (as during pancreas organogenesis ([31]–[32], C = Pdx1→Pax4→Mafa), in an opposite order (D = Mafa→Pax4→Pdx1) and in a random order (E = Pdx1→Mafa→Pax4) (Figure 3A). The effect of the sequential administration of pTFs on transdifferentiation efficiency and on the β-cell-like maturation was compared to that of the simultaneous administration of all three pTFs on the first day (B = Pdx1+Pax4+Mafa) and to similar MOI of control virus (β-gal = A, Figure 3A).

Figure 3 demonstrates that the transdifferentiation along the β-cell-like lineage was substantially, but similarly, increased by either concerted or sequential administration of pTFs under each of the analyzed orders (B–E, in Figure 3A). Insulin promoter activity (Figure S2A), the percent of insulin producing cells (Figure 3B) and glucose-regulated insulin (and or proinsulin) secretion (Figure 3C) remained unaffected by the order of the sequentially administered pTFs. Interestingly, the sequential administration of pTFs in the random order (E = Pdx1→Mafa→Pax4) resulted in increased insulin promoter activity, but was associated with loss of glucose regulation of insulin secretion and decrease of the expression of the glucose transporter 2; SLC2A2 (Figure 3C and Figure S2B). Loss of the glucose sensing ability upon changing the order of Pax4 and Mafa administration, may suggest a potential effect of the sequence of expressed pTFs on β cell-like maturation but not on the efficiency of the transdifferentiation process. Therefore, we analyzed to what extent and under which conditions, increased transdifferentiation efficiency is also associated with enhanced maturation along the β cell lineage.

### Enhanced Maturation of Transdifferentiated Liver Cells Along the β Cell Lineage Occurs Only Upon Direct Hierarchical Administration of Pdx1, Pax4 and Mafa

The hallmark characteristics of mature β cells are the capacity to process the proinsulin and secrete processed insulin in a glucose-regulated manner [41], [42]. To analyze whether the temporal changes in the expression of pTFs distinctly affect transdifferentiated cell maturation along the β cell lineage, we investigated the effect of the distinct treatments (A–E, Figure 3A) on proinsulin processing and glucose-regulated c-peptide secretion.

Indeed, only the sequential and *direct* hierarchical administration (C) of pTFs resulted in pronounced increased production of processed insulin. C-peptide secretion is glucose-regulated within the physiological range of glucose dose response characteristics (Figure 3D and Figure S3A). The newly acquired phenotype and function were stable, enabling us to follow glucose-regulated c-peptide secretion for at least four weeks *in vitro* (Figure S3B).

The increased prohormone processing induced by the direct hierarchical administration of pTFs (C) was associated with pronounced increase in gene expression of SLC2A2 and PCSK2 which possess roles in prohormone processing and glucose sensing abilities, respectively (Figure S2B&C).

These data suggest an obligatory role for the *sequential and direct* hierarchical expression of pTFs in promoting the maturation and function of the transdifferentiated liver cells along the β cell lineage. Both concerted and sequential administration of pTFs in an indirect hierarchical mode, failed to generate cells which display mature β-cell-like characteristics.

### The Sequence of the Ectopically Administered pTFs Affects the Profile of the Endogenously Activated pTFs in Liver Cells

To provide a mechanistic explanation to the distinct maturation outcome of the transdifferentiated cells, we analyzed the repertoire of the endogenously activated pTFs under the distinct temporal treatments (B–E, Figure 3A). All the protocols analyzed activated the expression of numerous endogenous pTFs (Figure 3). However, the most robust difference between the “mature” (C) and “immature” phenotypes (B, E and D, Figure 3A) was exhibited at the levels of the endogenous ISL1 gene expression at the end of the process, on the six day. Thus, the most enhanced maturation along the β-cell lineage induced by direct hierarchical administration of pTFs (C) correlates with a dramatic decrease in endogenous ISL1 expression (Figure 3E, arrow). Taken together, these data suggest that maturation of transdifferentiated liver cells along the β cells lineage correlates with a specific expression profile of pTFs. The diminished ISL1 expression only under the direct hierarchical C-protocol could be potentially related to the level of maturation of the transdifferentiated cells.

### The Individual Contribution of Each of the pTFs to Liver to Pancreas Transdifferentiation

**Figure 4:**
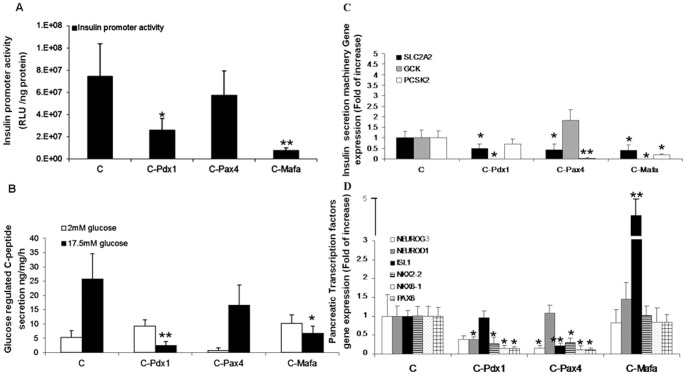
The individual role of the pancreatic transcription factors in the transdifferentiation process. Adult human liver cells were treated by the direct “hierarchical” sequential infection order (C, Figure 3A). One single transcription factor (pTF) was omitted at a time and replaced by an identical MOI of *Ad-CMV-β-gal*. Pdx1 omission is indicated as (C-Pdx1), Pax4 omission is indicated as (C-Pax4), and Mafa omission is indicated as (C-Mafa). **(A)** Insulin promoter activation analysis, results are presented mean ± SE, *p<0.1, **p<0.05 compared to the direct “hierarchical” sequential infection order (C). n≥6 in 3 independent experiments preformed in cells isolated from different donors. **(B)** C-peptide secretion was measured by static incubation for 15 min at 2 and 17.5 mM glucose in KRB. *p<0.05, **p<0.01 compared to the direct “hierarchical” sequential infection order (C). n≥6 in 3 independent experiments. (**C–D**) Quantitative Real-Time PCR analysis for the transcription levels of pancreatic enzymes **(C)** and pTFs **(D)**. CT values are normalized to β-actin gene expression within the same cDNA sample. Results are presented as relative levels of the mean±SE compared to “hierarchy sequential infection” treated liver cells. *p<0.05, **p<0.01, *n*≥6 in 3 independent experiments preformed in cells isolated from different donors.

We next analyzed the potential roles of ectopic pTFs in both inducing liver to pancreas transdifferentiation and promoting maturation of the newly generated cells along the β cell lineage. The separate contribution of each of the transcription factors to the hierarchical developmental process was analyzed by a relative and temporal “loss of function” approach. Adult human liver cells were treated by the sequential and direct hierarchical reprogramming protocol (C, in Figure 3A), from which one of the ectopic pTFs was omitted. The omitted pTF was replaced by a control adenovirus carrying β-gal expression at a similar multiplicity of infection. The functional consequences of separately omitting the expression of each of the pTFs were analyzed at the molecular and functional levels (Figure 4).

Pdx1 or Mafa omission (C-Pdx1 and C-Mafa, respectively) resulted in decreased insulin promoter activation (Figure 4A), ablated glucose response of processed insulin secretion (Figure 4B) and decreased SLC2A2 and GCK expression (Figure 4C). Mafa but not Pdx1 exclusion was associated also with decreased expression of the prohormone convertase, PCSK2 (Figure 4C). Pax4 exclusion (C-Pax4) did not significantly affect insulin promoter activation, but decreased SLC2A2 and PCSK2 expression.

Pdx1 and Pax4 exclusion caused a marked decline in the expression of most endogenously activated pTFs, suggesting their roles in promoting transdifferentiation efficiency via increasing the expression of most endogenous pancreatic TFs (Figure 4D). On the other hand, Mafa did not contribute to the increase in expression of the endogenous pTFs. On the contrary, its exclusion on the third day was associated with a robust increase of endogenous ISL1 expression (Figure 4D). These data may suggest that Mafa which affects the final stages of pancreas organogenesis is not involved in further promoting the efficiency of liver to pancreas transdifferentiation. Rather, it is potentially involved in promoting the maturation of the transdifferentiated cells in a process associated by ISL-1 repression.

### Isl1 Ablates Transdifferentiated Liver Cell Maturation Along the β Cell Lineage and Function

**Figure 5:**
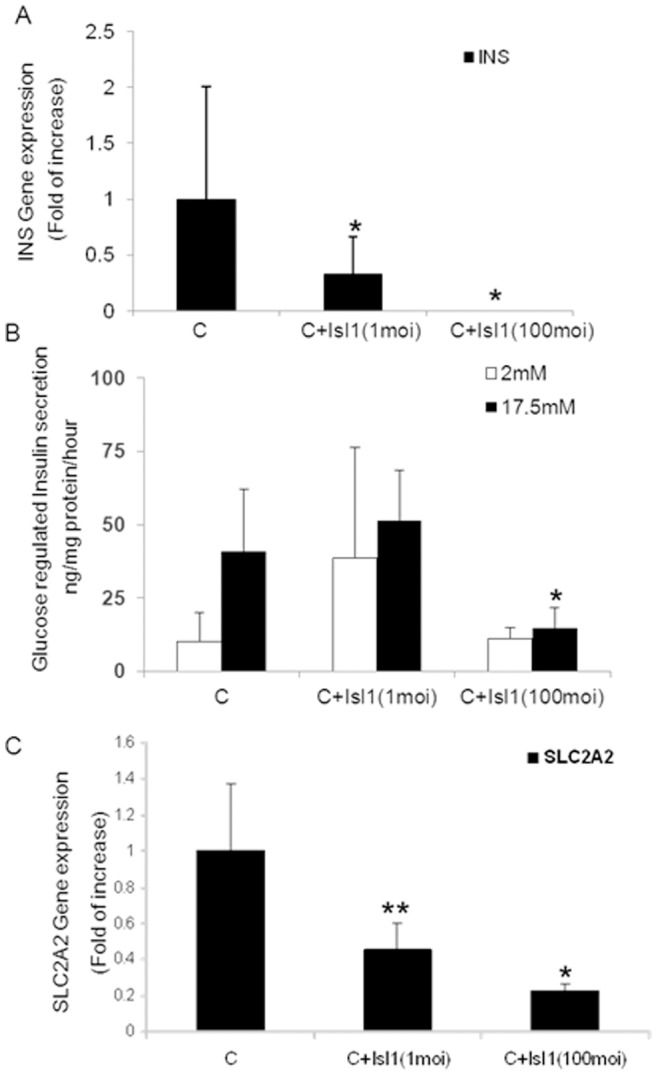
High Isl1 levels disrupt β cell maturation of transdifferentiated liver cells. Adult human liver cells were treated by the direct “hierarchical” sequential infection order (C) and supplemented by *Ad-CMV-Isl1* (1 or 100MOI) at the 3rd day (C+Isl1). **(A)** Quantitative Real-Time PCR analyses for insulin gene expression levels. CT values were normalized to β-actin gene expression within the same cDNA sample. Results are presented as relative levels of the mean ± SE compared to control virus treated cells. *P<0.05, n>6 in 3 independent experiments preformed in cells isolated from different donors. **(B)** Insulin (and or pro-insulin) secretion was measured by static incubation of the cells for 15 min at 2 and 17.5 mM glucose in KRB. **P<0.01, compared to the direct “hierarchical” sequential infection order (C). n>6 in 3 independent experiments preformed in cells isolated from different donors. **(C)** Quantitative Real-Time PCR analyses for the gene expression levels of SLC2A2. CT values are normalized to β-actin gene expression within the same cDNA sample. Results are presented as relative levels of the mean ±SE compared to control virus treated cells. *P<0.05, n>6 in 3 independent experiments preformed in cells isolated from different donors.

To analyze if and how ablated maturation of the transdifferentiated liver cells along the β cell lineage is related to increased Isl-1 expression, we ectopically introduced Isl1 (Ad- Isl1) into liver cells treated by sequential administration of pTFs in a direct hierarchical mode (treatment C, Figure 3A). When jointly administered with Mafa on the third day (C+Isl1), Isl1 substantially decreased insulin gene expression and ablated glucose-regulated insulin (and or proinsulin) secretion (Figure 5 A&B). Ectopic Isl1 expression in human pancreatic islets in culture displayed similar effects on insulin gene expression (data not presented) and on glucose regulated insulin secretion (supplement Figure 4). The loss of the glucose sensing ability was associated with diminished SLC2A2 expression (Figure 5C). These results suggest that increased Isl1 levels may indeed hamper the maturation of the transdifferentiated liver cells along the β cell lineage. Moreover, it suggests a role for Mafa in restraining Isl-1 levels and in promoting the maturation of the transdifferentiated cells along the β cell lineage and function.

### Increased Isl-1 Expression at the Final Stage of Transdifferentiation Results in the Generation of Insulin and Somatostatin Co-expressing Cells

**Figure 6:**
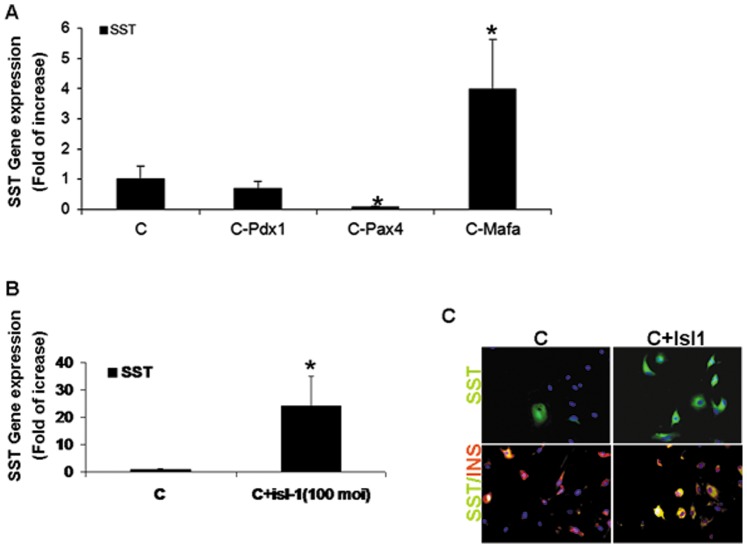
Over expression of Isl-1 at the end of the transdifferentiation process results in bi-hormonal cells expressing insulin and somatostatin. (A, B) Quantitative Real-Time PCR analysis for the pancreatic hormone Somatostatin. CT values are normalized to β-actin gene expression within the same cDNA sample. Results are presented as relative levels of the mean±SE compared to “hierarchy sequential infection” treated liver cells. *P<0.05, **P<0.1, n≥6 in 3 independent experiments preformed in cells isolated from different donors. (C) Immunofluorescence staining of treated human liver cells for Somatosatin (green) and Insulin (red). Nuclei were stained by DAPI (blue), original magnification X20.

Transdifferentiation along the endocrine pancreatic lineage results in the activation of numerous pancreatic hormones. Somatostatin gene expression was markedly increased in cells which were treated by the sequential protocol from which the ectopic Mafa expression was omitted (Figure 6A). We therefore analyzed whether the inhibitory effect of Mafa on somatostatin expression is related to its capacity to repress ISL1 expression. Indeed, Ad-Isl1 administration on the third day together with Mafa (C+Isl1) increased somatostatin gene expression (Figure 6B). Immunofluorescence analyses indicate that under these conditions, in addition to decreased insulin production and ablated response to glucose, most of the transdifferentiated cells stained positively to both somatostatin and insulin (Figure 6 C).

These results suggest that part of the maturation of the transdifferentiated β cells is attributed to Mafa expression at the final stage of the transdifferentiation process. At this stage, Mafa restricts somatostatin expression in a process associated with its capacity to directly or indirectly restrict ISL1 expression.

## Discussion

The hierarchical characteristics of the pTFs network that control pancreas organogenesis in the embryo have been elucidated *in vivo* by loss or gain of function studies [26], [43], [44]. Perturbations of the network hierarchy resulted in impaired pancreas development and improper sub-lineages segregation [30], [45]. The present study suggests that pancreatic endocrine cells characteristics generated by transdifferentiation of human liver cells rely on the hierarchical sequence of the ectopically introduced pTFs. Hence, despite being substantially faster than embryonic pancreas organogenesis [46], [47], transdifferentiation of liver to pancreas is a progressive and hierarchical process.

**Figure 7:**
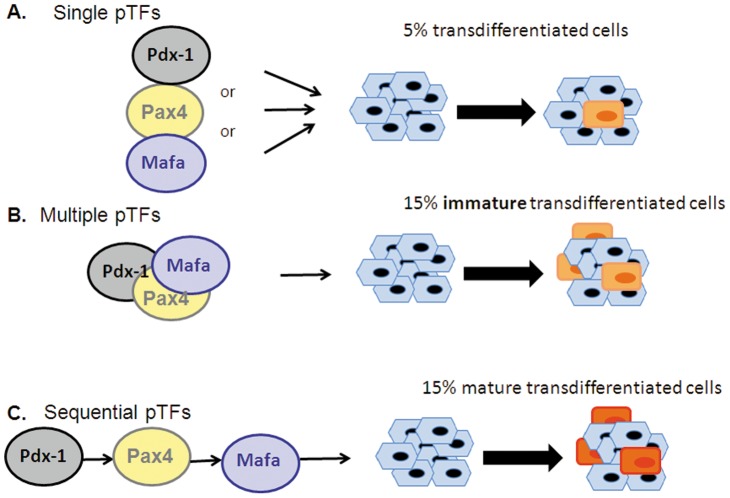
Schematic representation of the proposed sequential and hierarchical control of multiple pancreatic transcription factors (pTFs) induced liver to pancreas transdifferentiation. Liver to pancreas transdifferentiation is a fast and sequential process which is temporally controlled in a hierarchical manner. Three transcription factors promote the transdifferentiation efficiency such that many more cells produce pancreatic hormones (B compared to A). However, to increase both transdifferentiation efficiency and the cells maturation along the β cell lineage (maturation means pro-insulin processing and its glucose regulated secretion), the 3 pTFs which control distinct stages of pancreas organogenesis should be sequentially supplied in a direct hierarchical mode (C).

Our findings confirm that transdifferentiation efficiency is substantially increased by the combined ectopic expression of several pTFs over the use of individual ectopic TFs (Figure 2, [21], [39], [48]–[50]). However, it seems that co-expression of the 3 pTFs within the same cell is insufficient to activate the endocrine pancreatic repertoire. Only about half of the 29% of the cells which co-expressed all three ectopic pTFs stained positive to insulin (Figures 2, 3 & 7). This may suggest that the transdifferentiation protocol is still suboptimal or that it may be restricted to transdifferentiation-competent liver cells. Potential reprogramming competence has been suggested also in trans-conversion between other fates (8–12, 16).

Due to the progressive nature of the process (Figure 1), and due to the distinct roles of the individual pTFs in the process (Figure 4), only sequential pTFs administered in a direct hierarchical manner, results in the maturation of the transdifferentiated cells along the β cell lineage and function (Figure 3). Either simultaneous or scrambled sequential administration of the ectopic pTFs failed to achieve increased processing of the newly generated pro-insulin and its glucose-regulated secretion (Figure 3D).

The progressive nature of transdifferentiation allowed us to suggest individual roles of different pTFs in the process of liver to pancreas transdifferentiation, by “temporal” loss of function analysis (Figure 4). Pdx1 and Pax4 promote transdifferentiation via increasing the expression of numerous endogenous, otherwise silent, pTFs in the liver (Figure 4D). By contrast, Mafa which is expressed at final stages of endocrine pancreas development in the embryo did not further increase endogenous pTFs expression. Omission of Mafa from the last stage of transdifferentiation ablated the capacity of the transdifferentiated liver cells to mature into β-like-cells. This was manifested by ablated proinsulin processing and its glucose-regulated secretion (Figure 4). These findings are in agreement with the suggested role of Mafa in the maturation of pancreatic endocrine progenitor cells along the β cell lineage [30], [51]. Artificially preceding the expression of Mafa to E12.5 in transgenic mice embryos, hampered the generation and development of pancreatic endocrine cells [30]. Our data suggest a potential similar role for Mafa at the final stages of the transdifferentiation process; Mafa earlier ectopic expression, together with or prior to the expression of Pdx-1 and Pax-4 (Figure 3), hampered the maturation of the transdifferentiated cells along the β cells lineage. Our data further suggest a unique reciprocal association between Mafa and Isl-1 (Figure 4D). Treatments which lacked ectopic Mafa at the final stage of the process were characterized by increased endogenous ISL1 expression and the generation of immature β-like-cells (Figures 3E &4D). Indeed, Isl-1 over expression at the final stage of transdifferentiation ablated the maturation of the cells along the β cell lineage and function, despite the increased Mafa expression (Figure 5A–C). Isl-1 displayed a similar effect upon ectopic expression in human pancreatic islets (Figure S4). Taken together, we suggest that Isl-1 and Mafa may promote two opposing processes; Isl-1 may play a role in promoting the generation of endocrine precursor cells by increasing their proliferation [52], while Mafa may promote progenitor cells’ maturation [30]. Interestingly, Mafa has been identified as a direct positive transcriptional target of Isl-1 [52]. Our present findings may suggest a potential reciprocal involvement of Mafa in repressing the expression of Isl-1, once sufficient endocrine precursor cells are generated. Indeed, ectopic expression of Mafa in human pancreatic islets *in vitro* resulted in complete abolishment of endogenous Isl-1 gene expression (Figure S5). This hypothesis should be further analyzed in endocrine pancreatic progenitors. Mafa may only indirectly affect Isl-1 expression, since Mafa binding elements were not found on the Isl-1 promoter.

The temporal and hierarchical expression of pTFs is also known to selectively drive proper lineage specification and segregation within the endocrine pancreas during pancreas organogenesis [26], [53]. Using our *in vitro* experimental system, we demonstrate that transdifferentiated cells which display increased Isl-1 expression, are bi-hormonal, co-expressing both insulin and somatostatin (Figure 6B&C). Insulin and somatostatin co-expressing cells were found during pancreas organogenesis in the embryo but disappeared after birth [54]. Lineage tracing analyses performed *in vivo* suggest the potential segregation of insulin producing cells from glucagon or somatostatin co-expressing cells, in extreme environmental conditions [55], [56]. During liver to pancreas transdifferentiation, glucagon and somatostatin precede insulin expression (Figure 1), lineage tracing analyses may suggest whether and under which conditions β cells emerge from bi-hormonal precursors, during transdifferentiation. It has been recently suggested that NKX6.1 induces β-like maturation of transdifferentiated liver cells at the expense of glucagon producing cells [21].

In summary, we provide a novel insight of the transdifferentiation process, as schematically summarized in Figure 7. The efficiency of transdifferentiation is substantially increased by the combined expression of several lineage specific transcription factors (Figure 7A compared to 7B). However, due to the progressive nature of the process, only when the ectopic transcription factors are sequentially introduced in a direct hierarchical manner the increased transdifferentiation efficiency is translated also into increased maturation along the β cell lineage and function (Figure 7C).

Data generated using the liver to pancreas transdifferentiation experimental system suggest that the generated cell fates specification, segregation and maturation occur in a sequential manner and are controlled by temporal alterations in the relative expression of the endogenous pTFs. It will be interesting to analyze whether the characteristics of liver to pancreas transdifferentiation are general and could be possibly extended to improve the outcome of TFs-induced fate alterations between other adult tissues.

## Supporting Information

Figure S1 **Insulin producing cells express the ectopic pTFs.** Cultured adult human liver cells were infected with *Ad-CMV-Pdx1* (1000 MOI), *Ad-CMV-Pax4* (100 MOI) and *Ad-CMV-Mafa* (10 MOI), and pancreatic differentiation markers were examined six days later. Immunofluorescence co-staining of treated human liver cells for insulin (red) and pTFs (Pdx1(a) or Pax4 (b) or Mafa (c) in green). Nuclei were stained with DAPI (blue), original magnification X20. Arrows indicate the insulin and pTFs co-expression within the same cells.
(TIF)

Figure S2 **Both concerted and sequential expression of Pdx1, Pax4 and Mafa promote transdifferentiation efficiency.** Cultured adult human liver cells were infected with *Ad-CMV-Pdx1* (1000 MOI), *Ad-CMV-Pax4* (100 MOI) and *Ad-CMV-Mafa* (10 MOI) together or in a sequential manner as summarized in Figure 3A, and analyzed for their pancreatic differentiation six days later. **(A)** The cells were co-infected by the treatments with *Ad-RIP-LUC* (200 MOI), and the Luciferase activity is expressed as Relative Light Unit (RLU)/mg protein. Each data point represents the mean ± SE. *P<0.05, **P<0.01, n>4 in 2 independent experiments preformed in cells isolated from different donors. The significance represents the differences compared to control. **(B** and **C**) Quantitative Real-Time PCR analysis for SLC2A2 and PCSK2 gene expression, respectively. CT values are normalized to β-actin gene expression within the same cDNA sample. Results are presented as relative levels of the mean±SE compared to control virus treated cells. *P<0.05, n≥8 in 4 independent experiments preformed in cells isolated from different donors.
(TIF)

Figure S3 **Sequential expression of the pTFs in a direct hierarchical manner results in persistent secretion of processed insulin in physiological glucose concentrations.** Cultured adult human liver cells were treated by the direct “hierarchical” sequential order (C, in Figure 3A). (A) Glucose dose response of c-peptide secretion is performed by static incubation for 15 min at 0, 5, 10, 15, 20 mM glucose. *P<0.05, n≥7 in 3 independent experiments preformed in cells isolated from different donors. (B) Following the C protocol of transdifferentiation (C, in Figure 3A) the transdifferentiated cells were cultured for additional 13 or 28 days in serum free media supplemented with insulin, transferrin and selenium (ITS), before being analyzed for c-peptide secretion. *P<0.05, **P<0.01, n≥5 in 2 independent experiments preformed in cells isolated from different donors. The significance represents the differences compared to the standard protocol (C on day 6).
(TIF)

Figure S4 **High Isl1 levels hampers β cell maturation of isolated human pancreatic islets.** Human pancreatic islets were treated by *Ad-CMV-Isl1* (10 MOI). Insulin secretion was measured 5 days later by static incubation for 15 min at 2 and 17.5 mM glucose, *p<0.05, **p<0.01, n = 2 compared to untreated islets.
(TIF)

Figure S5 **Ectopic expression of Mafa decreases endogenous ISL-1 expression in isolated human pancreatic islets.** Human pancreatic islets were treated by *Ad-CMV-Mafa or by Ad-CMV-Pax4 (*both at 10MOI) and 5 days later analyzed by Quantitative Real-Time PCR for ISL1 gene expression levels. CT values are normalized to β-actin gene expression within the same cDNA sample. Results are presented as relative levels of the mean±SE compared to *Ad-CMV-Pax4* virus treated cells. *P<0.01, n≥3 in 2 independent experiments.
(TIF)

Table S1 **List of the primer sets used in this study.**
(DOC)

## References

[1] Atala A (2008) Extending life using tissue and organ replacement. Curr Aging Sci 1: 73–83. 20021376 10.2174/1874609810801020073

[2] Sheyn D, Mizrahi O, Benjamin S, Gazit Z, Pelled G, et al (2010) Genetically modified cells in regenerative medicine and tissue engineering. Adv Drug Deliv Rev 62: 683–698. 20114067 10.1016/j.addr.2010.01.002

[3] Slack JM, Tosh D (2001) Transdifferentiation and metaplasia–switching cell types. Curr Opin Genet Dev 11: 581–586. 11532402 10.1016/s0959-437x(00)00236-7

[4] Meivar-Levy I, Ferber S (2006) Regenerative medicine: using liver to generate pancreas for treating diabetes. Isr Med Assoc J 8: 430–434. 16833177

[5] Meivar-Levy I, Ferber S (2010) Adult cell fate reprogramming: converting liver to pancreas. Methods Mol Biol 636: 251–283. 20336528 10.1007/978-1-60761-691-7_16

[6] Russ HA, Efrat S (2011) Development of human insulin-producing cells for cell therapy of diabetes. Pediatr Endocrinol Rev 9: 590–597. 22397143

[7] Yechoor V, Chan L (2010) Minireview: beta-cell replacement therapy for diabetes in the 21st century: manipulation of cell fate by directed differentiation. Mol Endocrinol 24: 1501–1511. 20219891 10.1210/me.2009-0311 PMC2940465

[8] Ambasudhan R, Talantova M, Coleman R, Yuan X, Zhu S, et al (2011) Direct reprogramming of adult human fibroblasts to functional neurons under defined conditions. Cell Stem Cell 9: 113–118. 21802386 10.1016/j.stem.2011.07.002 PMC4567246

[9] Pang ZP, Yang N, Vierbuchen T, Ostermeier A, Fuentes DR, et al (2011) Induction of human neuronal cells by defined transcription factors. Nature 476: 220–223. 21617644 10.1038/nature10202 PMC3159048

[10] Vierbuchen T, Ostermeier A, Pang ZP, Kokubu Y, Sudhof TC, et al (2010) Direct conversion of fibroblasts to functional neurons by defined factors. Nature 463: 1035–1041. 20107439 10.1038/nature08797 PMC2829121

[11] Szabo E, Rampalli S, Risueno RM, Schnerch A, Mitchell R, et al (2010) Direct conversion of human fibroblasts to multilineage blood progenitors. Nature 468: 521–526. 21057492 10.1038/nature09591

[12] Ieda M, Fu JD, Delgado-Olguin P, Vedatham V, Hayashi Y, et al (2010) Direct reprogramming of fibroblasts into functional cardiomyocytes by defined factors. Cell 142: 375–386. 20691899 10.1016/j.cell.2010.07.002 PMC2919844

[13] Ber I, Shternhall K, Perl S, Ohanuna Z, Goldberg I, et al (2003) Functional, persistent, and extended liver to pancreas transdifferentiation. J Biol Chem 278: 31950–31957. 12775714 10.1074/jbc.M303127200

[14] Meivar-Levy I, Ferber S (2003) New organs from our own tissues: liver-to-pancreas transdifferentiation. Trends Endocrinol Metab 14: 460–466. 14643061 10.1016/j.tem.2003.10.006

[15] Kojima H, Fujimiya M, Matsumura K, Younan P, Imaeda H, et al (2003) NeuroD-betacellulin gene therapy induces islet neogenesis in the liver and reverses diabetes in mice. Nat Med 9: 596–603. 12704384 10.1038/nm867

[16] Sapir T, Shternhall K, Meivar-Levy I, Blumenfeld T, Cohen H, et al (2005) From the Cover: Cell-replacement therapy for diabetes: Generating functional insulin-producing tissue from adult human liver cells. Proc Natl Acad Sci U S A 102: 7964–7969. 15899968 10.1073/pnas.0405277102 PMC1142350

[17] Tang DQ, Lu S, Sun YP, Rodrigues E, Chou W, et al (2006) Reprogramming liver-stem WB cells into functional insulin-producing cells by persistent expression of Pdx1- and Pdx1-VP16 mediated by lentiviral vectors. Lab Invest 86: 83–93. 16294197 10.1038/labinvest.3700368 PMC3417286

[18] Wang AY, Ehrhardt A, Xu H, Kay MA (2007) Adenovirus Transduction is Required for the Correction of Diabetes Using Pdx-1 or Neurogenin-3 in the Liver. Mol Ther 15: 255–263. 17235302 10.1038/sj.mt.6300032

[19] Meivar-Levy I, Sapir T, Gefen-Halevi S, Aviv V, Barshack I, et al (2007) Pancreatic and duodenal homeobox gene 1 induces hepatic dedifferentiation by suppressing the expression of CCAAT/enhancer-binding protein beta. Hepatology 46: 898–905. 17705277 10.1002/hep.21766

[20] Aviv V, Meivar-Levy I, Rachmut IH, Rubinek T, Mor E, et al (2009) Exendin-4 promotes liver cell proliferation and enhances PDX-1-induced liver to pancreas transdifferentiation. J Biol Chem 284: 33509–33520. 19755420 10.1074/jbc.M109.017608 PMC2785195

[21] Gefen-Halevi S, Rachmut IH, Molakandov K, Berneman D, Mor E, et al (2010) NKX6.1 promotes PDX-1-induced liver to pancreatic beta-cells reprogramming. Cell Reprogram 12: 655–664. 21108535 10.1089/cell.2010.0030

[22] Meivar-Levy I, Sapir T, Berneman D, Weissbach T, Polak-Charcon S, et al.. (2011) Human liver cells expressing albumin and mesenchymal characteristics give rise to insulin-producing cells. J Transplant. 252387. 10.1155/2011/252387 PMC3163017 21876779

[23] Offield MF, Jetton TL, Labosky PA, Ray M, Stein RW, et al (1996) PDX-1 is required for pancreatic outgrowth and differentiation of the rostral duodenum. Development 122: 983–995. 8631275 10.1242/dev.122.3.983

[24] Stoffers DA, Thomas MK, Habener JF (1997) The homeodomain protein IDX-1. Trends Endocrinol & Metab 8: 145–151. 18406800 10.1016/s1043-2760(97)00008-8

[25] Gradwohl G, Dierich A, LeMeur M, Guillemot F (2000) neurogenin3 is required for the development of the four endocrine cell lineages of the pancreas. Proc Natl Acad Sci U S A 97: 1607–1611. 10677506 10.1073/pnas.97.4.1607 PMC26482

[26] Bernardo AS, Hay CW, Docherty K (2008) Pancreatic transcription factors and their role in the birth, life and survival of the pancreatic beta cell. Mol Cell Endocrinol 294: 1–9. 18687378 10.1016/j.mce.2008.07.006

[27] Brun T, Gauthier BR (2008) A focus on the role of Pax4 in mature pancreatic islet beta-cell expansion and survival in health and disease. J Mol Endocrinol 40: 37–45. 18234907 10.1677/JME-07-0134

[28] Collombat P, Mansouri A, Hecksher-Sorensen J, Serup P, Krull J, et al (2003) Opposing actions of Arx and Pax4 in endocrine pancreas development. Genes Dev 17: 2591–2603. 14561778 10.1101/gad.269003 PMC218152

[29] Kataoka K, Han SI, Shioda S, Hirai M, Nishizawa M, et al (2002) MafA is a glucose-regulated and pancreatic beta-cell-specific transcriptional activator for the insulin gene. J Biol Chem 277: 49903–49910. 12368292 10.1074/jbc.M206796200

[30] Nishimura W, Bonner-Weir S, Sharma A (2009) Expression of MafA in pancreatic progenitors is detrimental for pancreatic development. Dev Biol 333: 108–120. 19576197 10.1016/j.ydbio.2009.06.029 PMC2737322

[31] Habener JF, Kemp DM, Thomas MK, Gomez-Perez FJ, Mehta R (2005) Minireview: transcriptional regulation in pancreatic development Genetic defects of beta cell function: (MODY) application of molecular biology to clinical medicine. Endocrinology 146: 1025–1034. 15604203 10.1210/en.2004-1576

[32] Edlund H (2002) Pancreatic organogenesis–developmental mechanisms and implications for therapy. Nat Rev Genet 3: 524–532. 12094230 10.1038/nrg841

[33] Servitja JM, Ferrer J (2004) Transcriptional networks controlling pancreatic development and beta cell function. Diabetologia 47: 597–613. 15298336 10.1007/s00125-004-1368-9

[34] Seijffers R, Ben-David O, Cohen Y, Karasik A, Berezin M, et al (1999) Increase in PDX-1 levels suppresses insulin gene expression in RIN 1046–38 cells. Endocrinology 140: 3311–3317. 10385428 10.1210/endo.140.7.6796

[35] He TC, Zhou S, da Costa LT, Yu J, Kinzler KW, et al (1998) A simplified system for generating recombinant adenoviruses. Proc Natl Acad Sci U S A 95: 2509–2514. 9482916 10.1073/pnas.95.5.2509 PMC19394

[36] Kaneto H, Matsuoka TA, Nakatani Y, Miyatsuka T, Matsuhisa M, et al (2005) A crucial role of MafA as a novel therapeutic target for diabetes. J Biol Chem 280: 15047–15052. 15664997 10.1074/jbc.M412013200

[37] Tang DQ, Cao LZ, Chou W, Shun L, Farag C, et al (2006) Role of Pax4 in Pdx1-VP16-mediated liver-to-endocrine pancreas transdifferentiation. Lab Invest 86: 829–841. 16732298 10.1038/labinvest.3700434

[38] Song YD, Lee EJ, Yashar P, Pfaff LE, Kim SY, et al (2007) Islet cell differentiation in liver by combinatorial expression of transcription factors neurogenin-3, BETA2, and RIPE3b1. Biochem Biophys Res Commun 354: 334–339. 17239820 10.1016/j.bbrc.2006.12.216

[39] Zhou Q, Brown J, Kanarek A, Rajagopal J, Melton DA (2008) In vivo reprogramming of adult pancreatic exocrine cells to beta-cells. Nature 455: 627–632. 18754011 10.1038/nature07314 PMC9011918

[40] Varda-Bloom N, Shaish A, Gonen A, Levanon K, Greenbereger S, et al (2001) Tissue-specific gene therapy directed to tumor angiogenesis. Gene Ther 8: 819–827. 11423929 10.1038/sj.gt.3301472

[41] Borowiak M (2010) The new generation of beta-cells: replication, stem cell differentiation, and the role of small molecules. Rev Diabet Stud 7: 93–104. 21060968 10.1900/RDS.2010.7.93 PMC2989782

[42] Eberhard D, Lammert E (2009) The pancreatic beta-cell in the islet and organ community. Curr Opin Genet Dev 19: 469–475. 19713099 10.1016/j.gde.2009.07.003

[43] Chakrabarti SK, Mirmira RG (2003) Transcription factors direct the development and function of pancreatic b-cells. Trends Endocrinol Metab 14: 78–84. 12591178 10.1016/s1043-2760(02)00039-5

[44] Collombat P, Hecksher-Sorensen J, Serup P, Mansouri A (2006) Specifying pancreatic endocrine cell fates. Mech Dev 123: 501–512. 16822656 10.1016/j.mod.2006.05.006

[45] Habener JF, Kemp DM, Thomas MK (2005) Minireview: transcriptional regulation in pancreatic development. Endocrinology 146: 1025–1034. 15604203 10.1210/en.2004-1576

[46] Murtaugh LC, Melton DA (2003) Genes, signals, and lineages in pancreas development. Annu Rev Cell Dev Biol 19: 71–89. 14570564 10.1146/annurev.cellbio.19.111301.144752

[47] Bonal C, Herrera PL (2008) Genes controlling pancreas ontogeny. Int J Dev Biol 52: 823–835. 18956314 10.1387/ijdb.072444cb

[48] Kaneto H, Nakatani Y, Miyatsuka T, Matsuoka TA, Matsuhisa M, et al (2005) PDX-1/VP16 fusion protein, together with NeuroD or Ngn3, markedly induces insulin gene transcription and ameliorates glucose tolerance. Diabetes 54: 1009–1022. 15793239 10.2337/diabetes.54.4.1009

[49] Matsuoka TA, Kaneto H, Stein R, Miyatsuka T, Kawamori D, et al (2007) MafA regulates expression of genes important to islet beta-cell function. Mol Endocrinol 21: 2764–2774. 17636040 10.1210/me.2007-0028

[50] Song YD, Lee EJ, Yashar P, Pfaff LE, Kim SY, et al (2007) Islet cell differentiation in liver by combinatorial expression of transcription factors neurogenin-3, BETA2, and RIPE3b1. Biochem Biophys Res Commun 354: 334–339. 17239820 10.1016/j.bbrc.2006.12.216

[51] Aguayo-Mazzucato C, Koh A, El Khattabi I, Li WC, Toschi E, et al (2011) Mafa expression enhances glucose-responsive insulin secretion in neonatal rat beta cells. Diabetologia 54: 583–593. 21190012 10.1007/s00125-010-2026-z PMC3047400

[52] Du A, Hunter CS, Murray J, Noble D, Cai CL, et al (2009) Islet-1 is required for the maturation, proliferation, and survival of the endocrine pancreas. Diabetes 58: 2059–2069. 19502415 10.2337/db08-0987 PMC2731519

[53] Wilson ME, Scheel D, German MS (2003) Gene expression cascades in pancreatic development. Mech Dev 120: 65–80. 12490297 10.1016/s0925-4773(02)00333-7

[54] Vincent M, Guz Y, Rozenberg M, Webb G, Furuta M, et al (2003) Abrogation of protein convertase 2 activity results in delayed islet cell differentiation and maturation, increased alpha-cell proliferation, and islet neogenesis. Endocrinology 144: 4061–4069. 12933680 10.1210/en.2003-0088

[55] Courtney M, Pfeifer A, Al-Hasani K, Gjernes E, Vieira A, et al (2011) In vivo conversion of adult alpha-cells into beta-like cells: a new research avenue in the context of type 1 diabetes. Diabetes Obes Metab 13 Suppl 147–52. 10.1111/j.1463-1326.2011.01441.x 21824256

[56] Fernandes A, King LC, Guz Y, Stein R, Wright CV, et al (1997) Differentiation of new insulin-producing cells is induced by injury in adult pancreatic islets. Endocrinology 138: 1750–1762. 9075740 10.1210/endo.138.4.5049

