# Supplementary figures and images for: The Temporal and Hierarchical Control of Transcription Factors-Induced Liver to Pancreas Transdifferentiation
Source: PLoS One. 2014 Feb 4;9(2):e87812. doi: 10.1371/journal.pone.0087812 (PMC3913675; doi:10.1371/journal.pone.0087812)

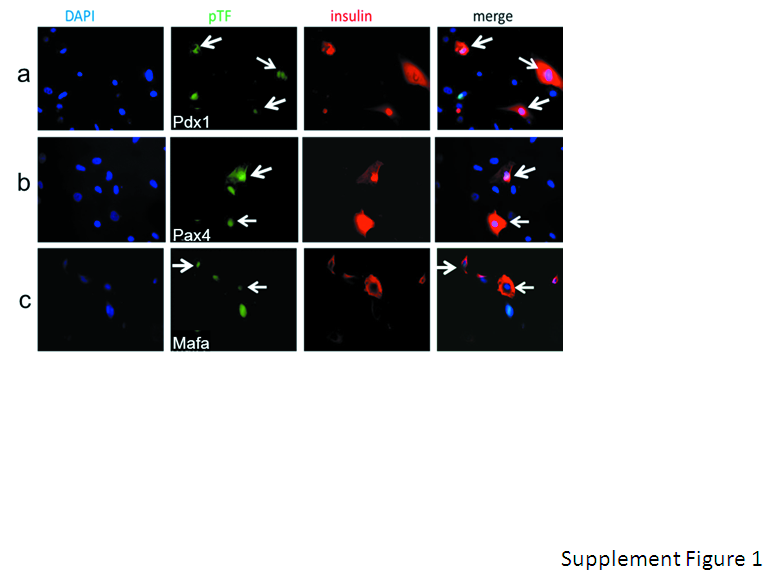

Supplement: Figure S1 — Insulin producing cells express the ectopic pTFs. Cultured adult human liver cells were infected with Ad-CMV-Pdx1 (1000 MOI), Ad-CMV-Pax4 (100 MOI) and Ad-CMV-Mafa (10 MOI), and pancreatic differentiation markers were examined six days later. Immunofluorescence co-staining of treated human liver cells for insulin (red) and pTFs (Pdx1(a) or Pax4 (b) or Mafa (c) in green). Nuclei were stained with DAPI (blue), original magnification X20. Arrows indicate the insulin and pTFs co-expression within the same cells. (TIF) [file pone.0087812.s001.tif]

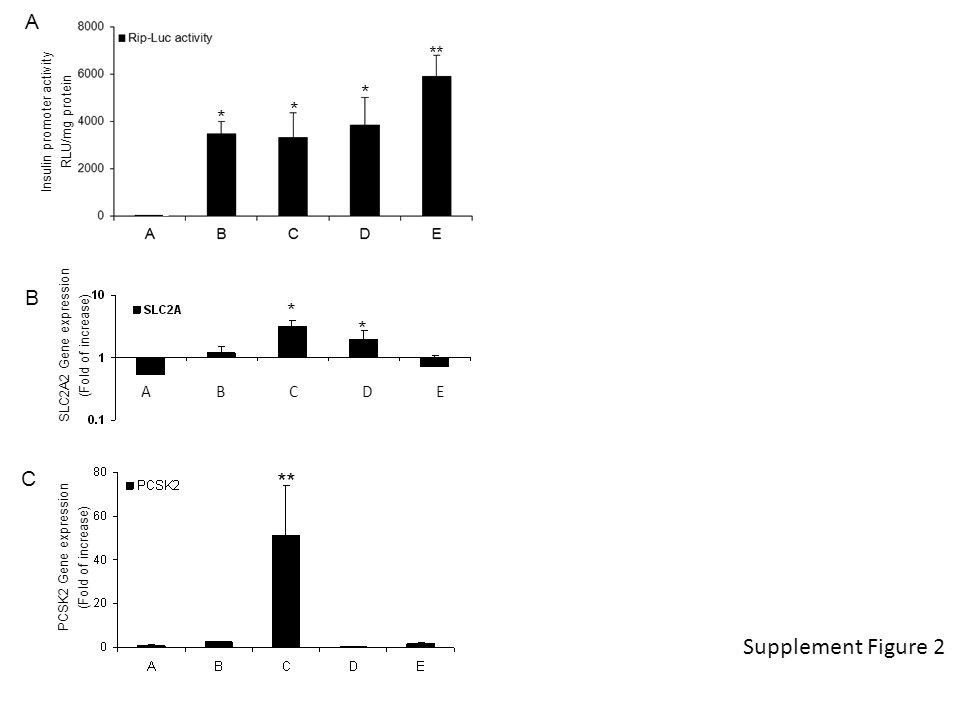

Supplement: Figure S2 — Both concerted and sequential expression of Pdx1, Pax4 and Mafa promote transdifferentiation efficiency. Cultured adult human liver cells were infected with Ad-CMV-Pdx1 (1000 MOI), Ad-CMV-Pax4 (100 MOI) and Ad-CMV-Mafa (10 MOI) together or in a sequential manner as summarized in Figure 3A, and analyzed for their pancreatic differentiation six days later. (A) The cells were co-infected by the treatments with Ad-RIP-LUC (200 MOI), and the Luciferase activity is expressed as Relative Light Unit (RLU)/mg protein. Each data point represents the mean ± SE. *P<0.05, **P<0.01, n>4 in 2 independent experiments preformed in cells isolated from different donors. The significance represents the differences compared to control. (B and C) Quantitative Real-Time PCR analysis for SLC2A2 and PCSK2 gene expression, respectively. CT values are normalized to β-actin gene expression within the same cDNA sample. Results are presented as relative levels of the mean±SE compared to control virus treated cells. *P<0.05, n≥8 in 4 independent experiments preformed in cells isolated from different donors. (TIF) [file pone.0087812.s002.tif]

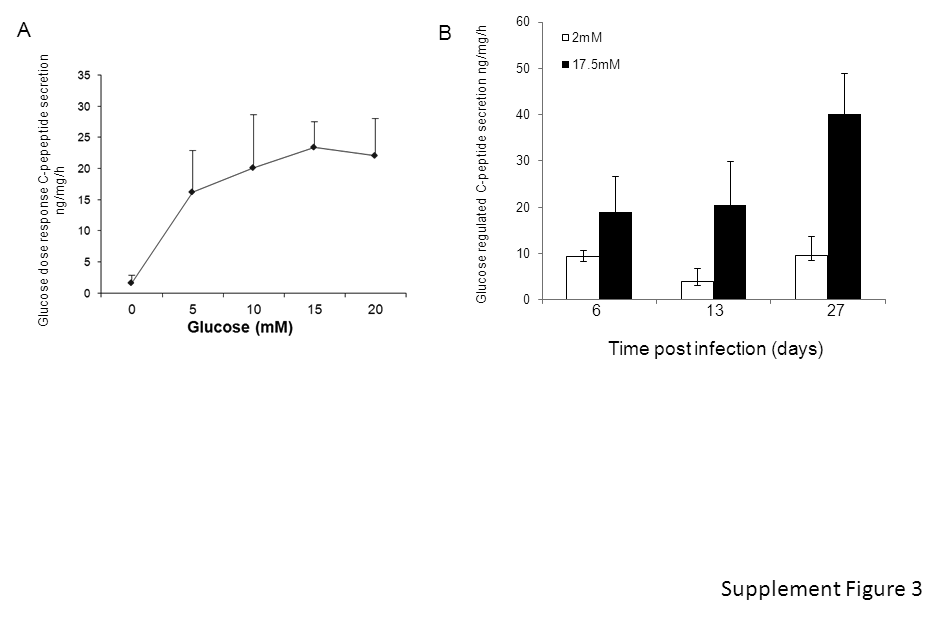

Supplement: Figure S3 — Sequential expression of the pTFs in a direct hierarchical manner results in persistent secretion of processed insulin in physiological glucose concentrations. Cultured adult human liver cells were treated by the direct “hierarchical” sequential order (C, in Figure 3A). (A) Glucose dose response of c-peptide secretion is performed by static incubation for 15 min at 0, 5, 10, 15, 20 mM glucose. *P<0.05, n≥7 in 3 independent experiments preformed in cells isolated from different donors. (B) Following the C protocol of transdifferentiation (C, in Figure 3A) the transdifferentiated cells were cultured for additional 13 or 28 days in serum free media supplemented with insulin, transferrin and selenium (ITS), before being analyzed for c-peptide secretion. *P<0.05, **P<0.01, n≥5 in 2 independent experiments preformed in cells isolated from different donors. The significance represents the differences compared to the standard protocol (C on day 6). (TIF) [file pone.0087812.s003.tif]

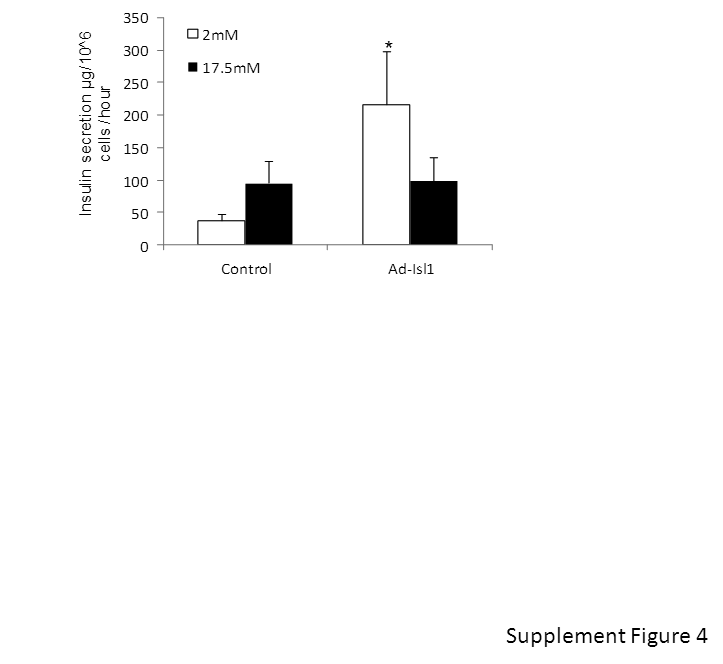

Supplement: Figure S4 — High Isl1 levels hampers β cell maturation of isolated human pancreatic islets. Human pancreatic islets were treated by Ad-CMV-Isl1 (10 MOI). Insulin secretion was measured 5 days later by static incubation for 15 min at 2 and 17.5 mM glucose, *p<0.05, **p<0.01, n = 2 compared to untreated islets. (TIF) [file pone.0087812.s004.tif]

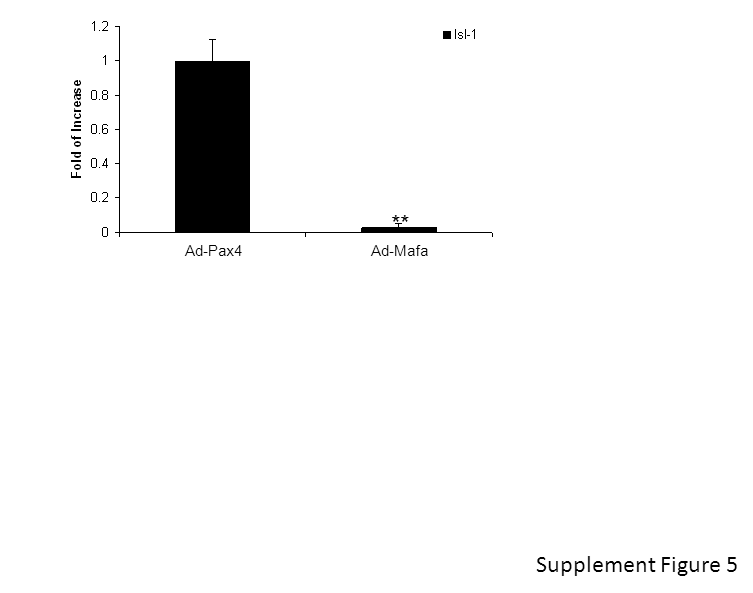

Supplement: Figure S5 — Ectopic expression of Mafa decreases endogenous ISL-1 expression in isolated human pancreatic islets. Human pancreatic islets were treated by Ad-CMV-Mafa or by Ad-CMV-Pax4 (both at 10MOI) and 5 days later analyzed by Quantitative Real-Time PCR for ISL1 gene expression levels. CT values are normalized to β-actin gene expression within the same cDNA sample. Results are presented as relative levels of the mean±SE compared to Ad-CMV-Pax4 virus treated cells. *P<0.01, n≥3 in 2 independent experiments. (TIF) [file pone.0087812.s005.tif]
