# Supplementary material for: The Temporal and Hierarchical Control of Transcription Factors-Induced Liver to Pancreas Transdifferentiation
Source: PLoS One. 2014 Feb 4;9(2):e87812. doi: 10.1371/journal.pone.0087812 (PMC3913675; doi:10.1371/journal.pone.0087812)
Supplement: Table S1 — List of the primer sets used in this study. (DOC) [file pone.0087812.s006.doc]

|  | F PRIMER | R PRIMER |
| --- | --- | --- |
| ARX | GCTGGGCCTGAGCACTTTC | AAAAGAGCCTGCCGAATGC |
| BRAIN4 |  |  |
| CGC | CCAAGATTTTGTGCAGTGGT | GGTAAAGGTCCCTTCAGCAT |
| GCK | CATCTCTGAGTGCATCTCCGACT | TCGCAGTGATGGTCTTCGTAGTA |
| GLUT2 | TCCAGCTACCGACAGCCTATT | CCAGCCGTCTGAAA AATGCT |
| INS | GCAGCCTTTGTGAACCAACA | CGGGTCTTGGGTGTGTAGAAGAAG |
| ISL1 | CGGGAGCCCTAATCCTCTCCCG | GCGGCGCAGCTGTTCTGATTA |
| MAFA | AGCAGCGGCACAT TCTGG | TTGTACAGGTCC CGCTCTTTG |
| NEUROD1 | ATGACCAAATCGTACAGCGAG | GTTCATGGCTTCGAGGTCGT |
| NEUROG3 | ACCCCATTCTCTCTTCTTTTCTC CT | GAGGCGTCATCCTTTCTACCG |
| NKX2-2 | GGC CTT CAGTAC TCC CT | GGG ACT TGG AGC TTG AGT CCT |
| NKX6-1 | CGTTGGGGATGACAGAGAGT | CGAGTCCTGCTTCTTCTTGG |
| PAX4 | CAGAGGCACTGGAGAAAGAGTTC | GGGCTTGAGACAGGCTTTAGG |
| PAX6 | TGCGACATTTCCCCAATTCT | GATGGAGCCAGTCTCGTAATACCT |
| PC1/3 | CTCTGGCTGCTGGCATCT | CTGCATATCTCGCCAGGTG |
| PCSK2 | GAGAAGACGCAGCCTACACC | CTGCAAAGCCATCTTTACCC |
| SST | ATGATGCCCTGGAACCTGAAG | GCCGGGTTTGAGTTAGCAGAT |

Table S1:
